# Comment on “The Use of Pulsed Electromagnetic Fields to Promote Bone Responses to Biomaterials* In Vitro* and* In Vivo*”

**DOI:** 10.1155/2019/2593205

**Published:** 2019-07-01

**Authors:** Raquel Ramirez-Vazquez, Isabel Escobar, Jesus Gonzalez-Rubio, Enrique Arribas

**Affiliations:** ^1^Applied Physics Department, Faculty of Computer Science Engineering, University of Castilla-La Mancha, Avda de España s/n, Campus Universitario, 02071 Albacete, Spain; ^2^Medical Sciences Department, School of Medicine, University of Castilla-La Mancha, C/ Almansa 14, 02071 Albacete, Spain

We have read the work of Galli [1] “*The Use of Pulsed Electromagnetic Fields to Promote Bone Responses to Biomaterials In Vitro and In Vivo”*, published the 3rd of September, 2018, in International Journal of Biomaterials, and we want to comment on some values of the magnetic fields used. In this publication, the authors present a review that includes studies investigating the effects of Pulsed Electromagnetic Fields (PEMFs) on the response of bone cells to different classes of biomaterials and the reports that focused on in vivo investigations of biomaterials implanted in bone.

In Tables 1, 2, and 3, on pages 3 and 6 to 8, the authors summarize the in vitro and in vivo studies on the effects of PEMFs stimulation on osteoblastic primary cells and cell lines on calcium phosphate biomaterials, titanium-based biomaterials, and polymer-based biomaterials, respectively. The data of magnetic field intensity are expressed in miliTesla (mT), except the field intensity of experimental model about placement in rabbit tibias (expressed in W). This last value is not considered for having the incorrect units.

We consider it interesting to do a detailed analysis of the average magnetic field used, to know their behaviour and calculate the intensity of the electromagnetic wave associated with this magnetic field. Supposing that the magnetic field is part of an electromagnetic wave, we have calculated the wave intensity of those waves using the data from Tables 1, 2 and 3, column 4, of the paper of Galli [1], obtaining the results of columns 3 and 6 (in italic font) of Table 1 (expressed in W/m^2^).

The expression we have used to calculate the intensity of the electromagnetic wave, measured in W/m^2^, is as follows:

(1) $$\begin{array}{c} I=\frac{c\cdot B_{max}^{2}}{{2\mu }_{0}} \end{array}$$

where* c* is the speed of light,* μ*_*0*_ is the magnetic permeability of the vacuum, and* Bmax* is the maximum value of the magnetic field applied to the tissue. Intensity is the power transferred per unit area (W/m^2^), where the area is transversal to the direction of propagation of the energy. Other authors call the intensity of the electromagnetic wave as energy flux, and it coincides with the Poynting vector module.

The calculated values of Table 1 are very large if we can compare them with the value of the solar radiation arriving at Earth from the Sun, known as the solar constant or the Total Solar Irradiance (TSI). TSI is the power per unit area measured above the Earth's atmosphere and normalized to the mean Sun–Earth distance of one AU (astronomical unit); the average value of this TSI is 1367 W/m^2^ [2, 3].

We would like to underline that this work has been very interesting for us, because the authors conclude that in these studies PEMFs have been repeatedly shown to possess the potential to affect osteoblast behavior on different biomaterials and thus represent a potential tool to improve the clinical outcome of several regenerative and prosthetic therapies; but if we compare the intensity of the electromagnetic wave calculated in W/m^2^ with the limits allowed by the International Commission on Nonionizing Radiation Protection (ICNIRP) [4], we observe that for PEMFs all of the values are too high. Therefore, we might conclude that magnetic field for PEMFs does not behave as part of electromagnetic waves for the calculation of wave intensity.

We have made some studies measuring personal exposition to radiofrequency electromagnetic fields (from 88 to 5850 MHz) [5, 6] in three different countries. In Table 2, we show the highest intensity registered during a period measured in each country and its corresponding magnetic field.

From the results of Table 2 we can obtain that they are within the limits established by INCIRP and, at the same time, they are much lower than the values in Table 1. Therefore, our conclusion is that Galli's values are enormous.

## Figures and Tables

**Table 1:** Studies on the effects of PEMF stimulation on osteoblastic primary cells and cell lines on calcium phosphate biomaterials, titanium-based biomaterials and polymer-based biomaterials, field intensity, and wave intensity.

Experimental model	Field intensity (mT)	*Wave Intensity (W/m* ^*2*^)	Experimental model	Field intensity (mT)	*Wave Intensity (W/m* ^*2*^)
Defects in proximal tibia of rabbits	0.18	*3.87·10* ^*6*^	Primary rat calvaria cells	0.96	*1.10·10* ^*8*^
Defects in rabbit tibia	8	*7.64·10* ^*9*^	Placement in rabbit tibias	0.4	*1.91·10* ^*7*^
Defects in rabbit femur (condyles)	1.6	*3.06·10* ^*8*^		-0.2	*4.77·10* ^*6*^
Defects in rabbit femurs (cortical bone, mid-diaphysis)	1.6	*3.06·10* ^*8*^	Murine MC3T3-E1 osteoblastic cells	2	*4.77·10* ^*8*^
Commercially available human mesenchymal stem cells	1.6	*3.06·10* ^*8*^	Defects in rabbit femurs (condyles)	2	*4.77·10* ^*8*^
Commercially available mesenchymal stem cells, normal human osteoblasts, MG-63 or Saos-2	1.6	*3.06·10* ^*8*^	Placement in rabbit femurs (condyles)	2	*4.77·10* ^*8*^
Human osteosarcoma Saos-2 cells	2	*4.77·10* ^*8*^	Human BMMSCs	2	*4.77·10* ^*8*^

Diaphysis of rabbit humerus	0.2	*4.77·10* ^*6*^	Human osteosarcoma MG-63 cells	2.3	*6.31·10* ^*8*^
Placement in rabbit femurs	0.2	*4.77·10* ^*6*^	Primary rat calvaria osteoblasts	0.13	*2.02·10* ^*6*^
	0.3	*1.07·10* ^*7*^		0.24	*6.88·10* ^*6*^
	0.8	*7.64·10* ^*7*^		0.32	*1.22·10* ^*7*^
Placement in rabbit mandibles	0.2	*4.77·10* ^*6*^	7F2+ RAW 264.7	1.5	*2.69·10* ^*8*^
Placement in tibias of ovariectomized rats	0.2	*4.77·10* ^*6*^	Human osteosarcoma Saos-2 cells	2	*4.77·10* ^*8*^
Human osteosarcoma Saos-2 cells	2	*4.77·10* ^*8*^	Osteochondral defects in rabbit medial femoral condyles	1.5	*2.69·10* ^*8*^
Human osteosarcoma Saos-2 cells	2	*4.77·10* ^*8*^	Rat calvaria defects	1	*1.19·10* ^*8*^
Placement in rat tibias	72	*6.19·10* ^*11*^	Human adipose tissue-derived stem cells	1	*1.19·10* ^*8*^
Dog mandibles, immediate postextraction placement	0.8	*7.64·10* ^*7*^			
Primary rat calvaria cells	0.2	*4.77·10* ^*6*^			

**Table 2:** The highest intensity measured in three different countries and its associated magnetic field.

Country	Electromagnetic Wave Intensity	Magnetic Field
	I (*µ*W/m^2^)	B(mT)
Spain	240.8	0.001420
Mexico	207.4	0.001318
Jordan	9826	0.009073
